# A Complicated Case of Group B Streptococcus Empyema Necessitans

**DOI:** 10.7759/cureus.1591

**Published:** 2017-08-22

**Authors:** Kevin G Buell, Saira Ajmal, Jennifer A Whitaker

**Affiliations:** 1 School of Medicine, Imperial College London; 2 Division of Infectious Diseases, Mayo clinic

**Keywords:** empyema necessitans, septic arthritis, pelvic abscess, group b streptococcus

## Abstract

Empyema necessitans is a complication of a pleural space infection that dissects through the pleura into the soft tissues of the chest and skin. Due to the widespread availability of antibiotics, empyema necessitans is rare in modern medicine and is most commonly caused by Mycobacterium tuberculosis*. *A 38-year-old immunocompetent male presented with left shoulder pain and his chest radiograph revealed a mass in the upper left lung and/or pleural space. He underwent multiple debridements of the chest wall due to a left chest wall abscess and empyema necessitans. All operative samples were positive for Streptococcus agalactiae (Group B Streptococcus). The patient’s clinical course was complicated by septic arthritis of the left sternoclavicular joint and first rib, vertebral osteomyelitis/discitis, and pelvic abscesses. This case report illustrates the pathogenic process of empyema necessitans and summarizes the clinical management for practicing clinicians. It also documents the second case of Streptococcus agalactiae-associated empyema necessitans, to our knowledge, with significantly greater disease extension than the first.

## Introduction

Lower respiratory tract infections can cause increased interstitial fluid production and pleural effusions. An empyema can subsequently develop due to inflammation in the pleural space, resulting in the formation of turbid fluid containing pus. Rarely, this process can be complicated by the purulent material dissecting through the pleura into the soft tissues of the chest and skin [1]; this is known as an empyema necessitans. The case report we present illustrates this pathogenic process, documents an unusual causative microorganism, and summarizes the clinical management of empyema necessitans for practicing clinicians.

## Case presentation

A 38-year-old gentleman, with no significant past medical history, consulted his primary care physician due to severe pain in the left shoulder. He had been treated for sinusitis three months prior with a three-day course of prednisone, and oral amoxicillin-clavulanic acid after presenting with 10 days of a productive cough.

**Figure 1:**
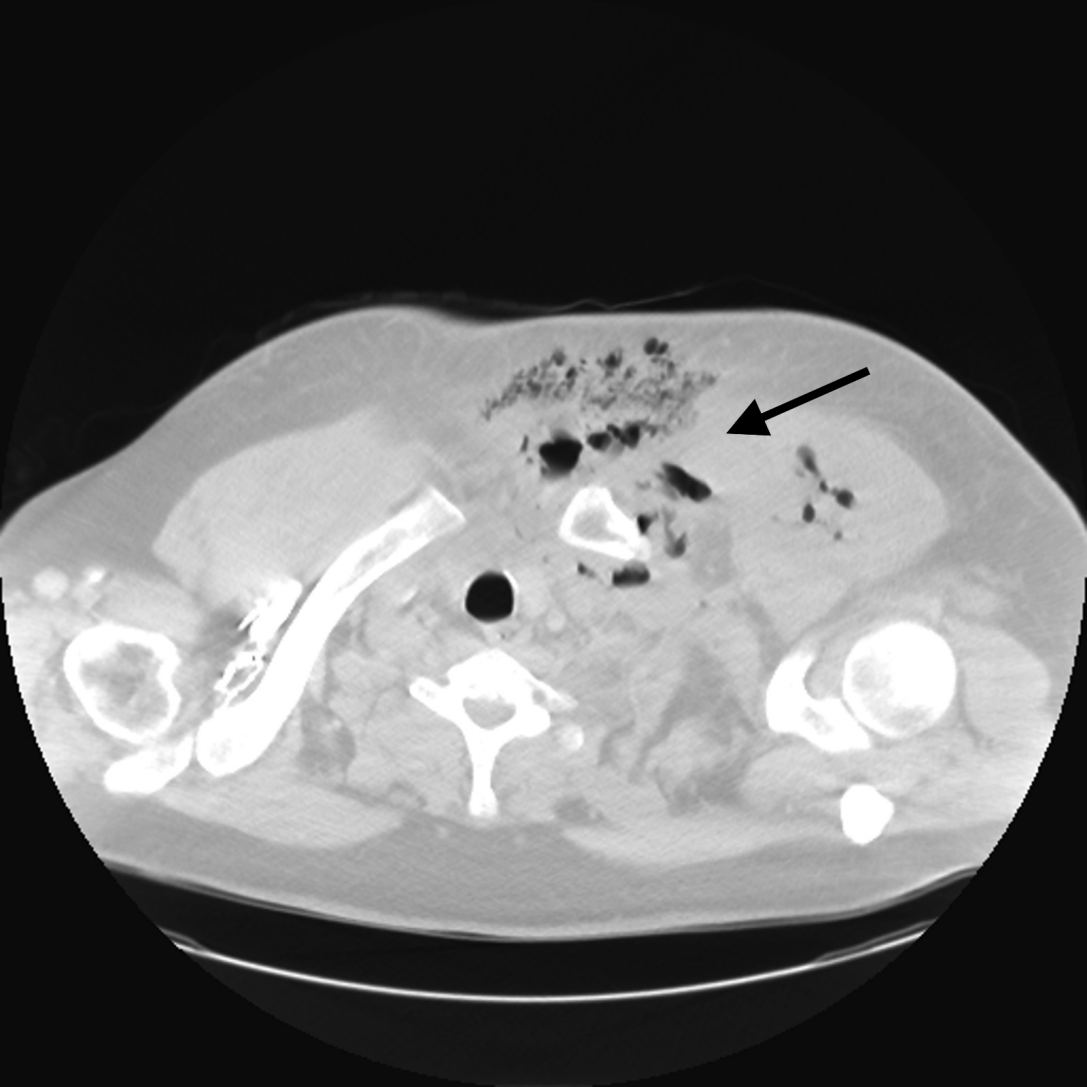
Chest computed tomography with intravenous contrast demonstrating chest wall collection extending into the anterior mediastinum

On examination, the patient was found to be febrile with a temperature of 38.2°C (100.8°F), tachycardic (122 beats per minute), and tachypneic (28 breaths per minute). His oxygen saturation on room air and blood pressure were within normal limits. He was noted to have a swelling in the left anterior chest wall that distorted the shape of his left shoulder. The swelling was firm, tender to palpation, and had overlying erythematous skin changes. On auscultation, there were coarse bibasilar crackles and decreased breath sounds bilaterally. The rest of the examination was unremarkable. Laboratory testing revealed normocytic anemia with hemoglobin of 8.5 g/dl and leukocytosis with a white blood cell count of 23.8 x 10^9^/L with a neutrophil predominance of 18.3 x 10^9^/L. A chest radiograph described a mass in the upper left lung and/or pleural space. A chest computed tomography (CT) scan with intravenous (IV) contrast characterized a 3.1 x 2.3 inches cavity on the anterior side of the lung apex that abutted to the costal pleura and a small portion of the mediastinal pleura. There were gas and fluid that extended from the cavity into the anterior mediastinum and left chest wall to just under the skin. Mild ground-glass infiltrates were also present around the left upper lung cavity (Figure 1).

A debridement of the chest wall, as opposed to CT-guided aspiration, was performed for source control due to the large size and possible extension of the cavity into the skin as visualized on CT. Intraoperatively, there was pus from the cavity under the left pectoralis muscle, consistent with a left chest wall abscess cavity and empyema necessitans. Operative samples were sent to pathology and microbiology, and the wound was covered with a wound vacuum-assisted closure device. The patient was started on empiric vancomycin and piperacillin/tazobactam for broad spectrum coverage against gram-positive organisms including methicillin-resistant Staphylococcus aureus (MRSA), gram-negative, and anaerobic organisms.

Cultures from the urine, sputum, and thoracic fluid collection all grew Streptococcus agalactiae, also known as Group B Streptococcus (GBS); blood cultures were negative. Susceptibility testing revealed susceptibility to penicillin G (minimum inhibitory concentration (MIC) ≤ 0.06), ampicillin (MIC ≤ 0.25), clindamycin (MIC ≤ 0.25), vancomycin (MIC = 0.5), and resistance to erythromycin (MIC = 2). He was de-escalated to piperacillin/tazobactam monotherapy, given that he had been on this antibiotic at the time of debridement and the possibility that this could have influenced culture results. Tuberculosis was ruled out with negative tissue and fluid acid-fast smears and mycobacterial cultures (held for 60 days), tissue Mycobacterium tuberculosis complex polymerase chain reaction (PCR), and QuantiFERON®-TB Gold In-Tube test (Cellestis Limited, Carnegie, Victoria, Australia). In addition, invasive fungal disease was excluded with negative tissue and fluid fungal smears and cultures (held for 24 days), fungal histopathology, tissue Histoplasma/Blastomyces PCR, and serum cryptococcal antigen. Tissue actinomyces cultures (held 14 days) and human immunodeficiency virus (HIV) serology were negative as well.

The patient underwent four further surgeries for washout of the left chest wall with debridement of the sternoclavicular joint and first rib due to cortical bone destruction and osteomyelitis. After initial improvement, the patient had recurrent fevers two weeks into his admission and developed lower back pain with associated left foot weakness. On examination, there was reduced dorsiflexion and eversion of the left foot with decreased sensation to pinprick in the L5 distribution. The rest of the neurological examination was normal, including rectal tone, perianal sensation, and fecal-urinary function. Inflammatory markers were elevated (C-reactive protein at 113 mg/dL and an erythrocyte sedimentation rate of 113 mm/hr). Magnetic resonance imaging (MRI) demonstrated discitis/osteomyelitis at L5-S1 and three abscesses: a 0.47-inch paraspinal abscess posterior to L5, a 1.1-inch abscess in the pelvis lateral to the left psoas muscle, and a 0.6-inch abscess within the left psoas muscle. The abscesses were not considered suitable for surgical drainage, and the patient was treated medically with piperacillin/tazobactam.

Three weeks into his hospitalization, the patient underwent closure of the chest wall with reconstruction using a left pectoralis flap. He was subsequently discharged on a four-week course of intravenous ertapenem for outpatient antibiotic therapy. Ertapenem provided a similar spectrum of antimicrobial coverage with the additional benefit of once daily, as opposed to four times daily infusions. At follow-up eight weeks into therapy, he was doing well clinically and inflammatory markers were trending down (C-reactive protein 23 mg/L, erythrocyte sedimentation rate 18 mm/hr); however, as the inflammatory markers were still mildly elevated, he was prescribed four-weeks of oral amoxicillin, with plans for infectious diseases follow-up and repeat evaluation of inflammatory markers. The patient did not return to infectious diseases; however, at a plastic surgery follow-up, he was noted to be doing clinically well off antibiotics one month after completing his amoxicillin.

## Discussion

As illustrated by our case report, empyema necessitans can present with pleuritic chest pain, nonproductive cough, and bulging of the anterior chest wall. Although the presence of crepitus and caseating discharge over a soft tissue mass may guide clinicians into performing early diagnostic imaging, empyema necessitans can misleadingly lack the traditional features of inflammation (erythema, warmth, and pain) [2]. Chest radiography is commonly the first imaging study performed to accurately identify thoracic fluid collections; yet, the preferred imaging modality is CT. It provides further diagnostic information, identifies associated lymphadenopathy, and accurately distinguishes between lung abscesses, cavitary lesions, and empyema. CT can additionally demonstrate the spread of the empyema necessitans into neighboring structures with previous case studies describing invasion into both extra and intra-thoracic structures, such as the bronchus, vertebrae, diaphragm, mediastinum, and retroperitoneum organs [3].

Pleural fluid should be obtained through thoracocentesis, chest tube thoracostomy, or surgical drainage of the collection. Sample fluid should be analyzed by cytology to exclude an underlying neoplastic process (such as lymphoma, bronchogenic carcinoma, and mesothelioma), biochemistry to demonstrate the exudative nature of the fluid, and microbiology for mycobacterial, bacterial, and fungal cultures. The treatment goal of empyema necessitans is to control the pleural infection through drainage of purulent material and antibiotic therapy. A case-by-case approach based on the clinical situation should be undertaken to reach this goal. Local antibiotic guidelines and susceptibility testing should guide the choice of antibiotic therapy. Surgical input, such as video-assisted thoracoscopic surgery, open drainage, decortication, or surgical removal of a lobe or lung, may be required in severe cases [4].

The most common infectious agent cultured is Mycobacterium tuberculosis, although Actinomyces sp., Staphylococcus aureus, Fusobacterium sp., and Streptococcus milleri have also been described [5]. To our knowledge, there has only been one previously documented case of GBS empyema necessitans [6]. Although GBS was previously known for epidemic outbreaks before the pasteurization of milk, today it has become synonymous with the colonization of the female genital tract, with a potential for secondary neonatal sepsis and meningitis. However, its role in adult non–gynecological diseases, such as soft tissue infection and pneumonia, is poorly understood despite a rising prevalence of disease. The most common clinical picture remains GBS-associated bloodstream infection without an identifiable source [7]. It occurs almost exclusively in patients with underlying medical comorbidities: diabetes mellitus, immunosuppression (immunosuppressive therapy, HIV, asplenia, solid organ transplant recipients), malignancy, lung disease, smoking, and liver disease. GBS strains are commonly penicillin, ampicillin, and vancomycin-susceptible with emerging resistance to erythromycin and clindamycin. Consequently, the treatment of choice remains penicillin-derived antibiotics [7].

## Conclusions

Due to the widespread availability of antibiotics, empyema necessitans is rare in modern medicine. Although several case reports are described in the scientific literature, there has yet to be a comprehensive systematic review. Our case report demonstrated the invasive capacity of GBS, with the most likely source of infection being his recent sinusitis. The patient’s community-acquired infection was subsequently complicated by the development of pneumonia, empyema, empyema necessitans, septic arthritis, vertebral osteomyelitis/discitis, and pelvic abscesses. The patient had no predisposing factors for GBS infection. We would like to caution the medical profession of the pathogenic potential of GBS outside gynecological and pediatric infections by documenting the second case of GBS-associated empyema necessitans in the literature.
